# Potassium and Humic Acid Synergistically Increase Salt Tolerance and Nutrient Uptake in Contrasting Wheat Genotypes through Ionic Homeostasis and Activation of Antioxidant Enzymes

**DOI:** 10.3390/plants11030263

**Published:** 2022-01-19

**Authors:** Ghulam Abbas, Sadia Rehman, Manzer H. Siddiqui, Hayssam M. Ali, Muhammad Ansar Farooq, Yinglong Chen

**Affiliations:** 1 Department of Environmental Sciences, COMSATS University Islamabad, Vehari Campus, Vehari 61100, Pakistan; sadiaguria911@gmail.com; 2 Department of Botany and Microbiology, College of Science, King Saud University, Riyadh 11451, Saudi Arabia; mhsiddiqui@ksu.edu.sa (M.H.S.); hayhassan@ksu.edu.sa (H.M.A.); 3 Institute of Environmental Sciences & Engineering, School of Civil & Environmental Engineering, National University of Sciences & Technology (NUST), Islamabad 44000, Pakistan; ansar@iese.nust.edu.pk; 4 The UWA Institute of Agriculture, UWA School of Agriculture and Environment, The University of Western Australia, Perth, WA 6001, Australia; 5 Institute of Soil and Water Conservation, Northwest A&F University, Yangling, Xianyang 712100, China

**Keywords:** salinity, potassium, humic acid, ROS, antioxidants

## Abstract

Salinity limits the growth and nutrient uptake in crop species. Studies show that both potassium (K) and humic acid (HA) improved plant tolerance to salinity. However, the interactive effect of K and HA on plant tolerance to salinity stress remains unknown. This pot study examined the effect of application of K (0, 5 or 10 mM) and HA (0 or 2 g kg^−1^), alone or in combination, on the growth and physiology under salinity (100 mM NaCl) in two wheat genotypes (SARC 1, salt tolerant; and SARC 5, salt sensitive). The results revealed that salt stress reduced shoot biomass by 35% and 49% in SARC 1 and SARC 5, respectively. Salinity induced overproduction of H_2_O_2_ and lipid peroxidation in both genotypes, but the decline in pigments and stomatal conductance was more profound in SARC 5 than in SARC 1. Combined application of 10 mM K and HA was most effective in alleviating salt stress with improved plant biomass by 47% and 43% in SARC 1 and SARC 5, respectively. Combined application of 10 mM K and HA mitigated salt and induced oxidative stress with the activities of APX, CAT, POD and SOD increased by up to 2.8 folds in SARC 1, and by upto 2.5 folds in SARC 5, respectively. Root and shoot Na contents were increased, while K, Fe and Zn contents were decreased under saline conditions. HA combined with K decreased Na and increased K, Fe and Zn contents in both genotypes. Combined application of 10 mM K and HA was more promising for increasing wheat salt tolerance and nutrient uptake and genotype SARC 1 performed better than SARC 5 for cultivation on saline soils.

## 1. Introduction

Increase in soil salinization is posing serious threat to crop productivity worldwide, predominantly in arid and semi-arid areas like Pakistan and northern China [1,2,3]. Approximately, 953 million hectares (mha) of the arable land across the globe (8% of the global land area) are affected by soil salinization to variable extents [4]. In Pakistan as an example, there is a continuous increase in the land degradation by soil salinity that has reached 10 mha accounting for ~12.9 % of the total country’s land [5].

The presence of excessive soluble salts in the rhizosphere interferes with normal plant physiological and metabolic functions and consequently translate into compromised plant growth and yield related attributes [3,6]. For instance, salt-induced osmotic stress reduces plant water uptake potential coupled with impaired uptake of essential plant nutrients. These effects are accompanied by specific ion toxicity due to enhanced uptake and accumulation of sodium (Na^+^) and chloride (Cl^−^) ions [7,8,9,10]. Furthermore, plant exposure to excessive salinity also results in the formation of reactive oxygen species (ROS), such as hydroxyl radical (·OH), superoxide (O_2_^−^), and hydrogen peroxide (H_2_O_2_) in several plant organelles, e.g., chloroplasts, peroxisomes and mitochondria [11]. Excessive ROS production and accumulation damages cellular constituents, such as nucleic acids, lipids, proteins as well as the integrity of cellular membranes [12]. To counteract ROS-induced oxidative damage, plants deploy various antioxidant enzymes including ascorbate peroxidase (APX), catalase (CAT), peroxidase (POD) and superoxide dismutase (SOD) that help crop plants to cope with multiple environmental stresses including salinity stress [13,14].

Supplementation with various mineral nutrients and organic amendments have been used to increase the salt tolerance potential of different crop plants [9,11]. Among these, potassium (K) as a macronutrient is essential for plants as it is involved in various biochemicals and physiological processes in plant growth and development [15]. It has indispensable role in enzymatic activation, protein synthesis, photosynthesis, stomatal regulation, cation-anion balance, energy transmission and osmoregulation etc., particularly under salinity stress [16,17,18]. Better uptake of K ions over Na is an important determinant of salt tolerance ability of plants [1,7]. It has been proposed that K is more efficient for osmotic adjustment than either Na or Cl under saline conditions [19,20]. The availability of K for plants is drastically decreased under saline conditions [21]. Hence, adequate amount of K within the cells is necessary to offset the damaging effects of salt stress [18].

Among organic amendments, humic acid (HA), an important component of humic substances—has demonstrated to play crucial role in increasing plant tolerance against several environmental stresses including drought and salinity [22,23,24,25]. Humic acid improves crop salt tolerance through the activation of numerous physiological and biochemical processes, such as improved water relations, stomatal conductance, and activation of the antioxidant enzymes (CAT, POD and SOD) to counter the deleterious effects of ROS [25,26]. It decreases peroxidation and increases the total protein contents as well as improves uptake of essential plant nutrients, particularly under marginal soil conditions [27,28,29].

Wheat (*Triticum aestivum* L.) is a dietary staple and exhibit great genotypic variation in the presence of rhizosphere salinity. This variation is mainly dependent on the extent and interval of salt stress [30,31]. Additionally, there is a great genetic variability among wheat genotypes regarding their response to salt stress. This genetic variability has been explored so some extent [1]. However, under saline conditions, in addition to ion toxicity, plants also face deficiency of many essential nutrients [1,6]. Both K and HA have shown promising results in increasing salt tolerance and nutrient uptake in different plant species. For example, supplementation of K under salinity stress increased grain yield and plant tolerance to salinity in wheat by restricting the uptake of Na, while increasing uptake of essential nutrients, better metabolism of nitrogenous compounds, improved plant water relations and photosynthetic attributes, and regulation of the ascorbate–glutathione cycle [7,17]. On the other hand, HA has been reported to increase salt tolerance and biomass production of plants by decreasing the generation of ROS [25]. It is involved in various abiotic stress-related genes encoding such as heat-shock proteins and redox proteins genes, such as HSP101, HSP23.6, HSP81.1, HSP17.6 and HSP26.5 [32]. Moreover, it has been reported to increase the nutrient availability for plants by lowering the soil pH [29]. However, the combined application of K and HA for enhancing salt tolerance and nutrient uptake capacity in wheat has not been considered yet. Therefore, the present study aimed to determine the interactive effects of K and HA for improving salinity tolerance and nutrient uptake in two wheat genotypes with contrasting salt tolerance.

## 2. Results

### 2.1. Plant Growth

**Figure 1:**
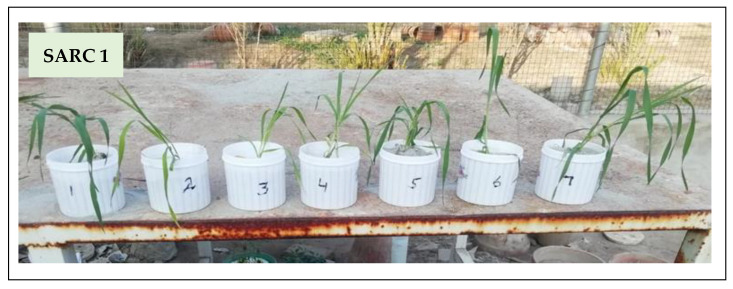

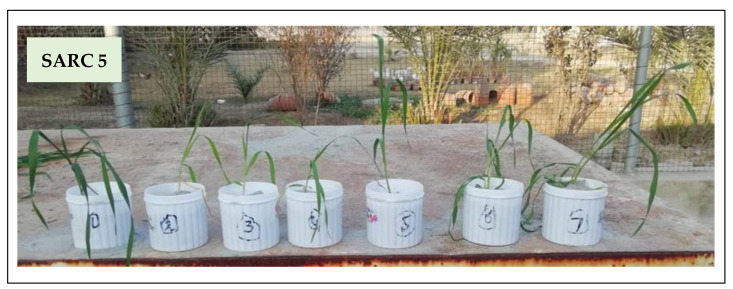
Effect of various treatments: 1; control, 2; salinity, 3; salinity + 5K, 4; salinity + 10K, 5; salinity + HA, 6; salinity + 5K+ HA, 7; salinity + 10K+ HA on growth of salt tolerant (SARC 1) and salt sensitive (SARC 5) wheat genotypes.

Shoot and root growth of both genotypes decreased under salinity stress. The application of K and its combination with HA relieved the salt stress in both genotypes (Table 1; Figure 1). Shoot length of SARC 1 and SARC 5 was decreased by 34% and 43% under salinity in comparison to control. The application of 10 mM K to the salt stress plants produced 20% and 15% increase in shoot length of both genotypes compared to salt stress. However, the combined application of 10 mM K and HA was the most effective treatment and it caused 48% and 33% increase in shoot length of SARC 1 and SARC 5, respectively as compared to salt stress (Figure 1). Root length of SARC 1 and SARC 5 decreased by 35% and 44% under salinity. Potassium supplementation (10 mM) caused 20–25% increase in root length of both genotypes with respect to salinity treatment. The highest increase in root length (48% and 36%) of SARC 1 and SARC 5, respectively was noticed under the combined application of 10 mM K and HA. Shoot dry weight was decreased by 35% and 49% under salinity and was improved by 47% and 43% under the combined application of 10 mM K and HA in SARC 1 and SARC 5, respectively. Root dry weight was decreased by 33% and 46% under salinity as compared to control. The combined application of 10 mM K and HA caused 50% and 38% increase in root dry weight of SARC 1 and SARC 5, respectively as compared to salt stress.

### 2.2. Stomatal Conductance and Chlorophyll Contents

Stomatal conductance and chlorophyll contents of both genotypes decreased under salinity stress. Salt stress caused approximately 20% and 30% reduction in stomatal conductance and photosynthetic pigments of SARC 1 and SARC 5 genotypes (Table 2). The combined application of 10 mM K and HA to SARC 1 caused 25%, 39%, 37% and 38% increase in stomatal conductance and Chl-a, Chl-b and total-Chl contents, respectively, as compared to salt stress. The respective increase in these attributes in the case of SARC 5 was 22%, 36%, 33% and 34%, respectively than salt stress.

### 2.3. Nutrient Element Measurements

**Figure 2:**
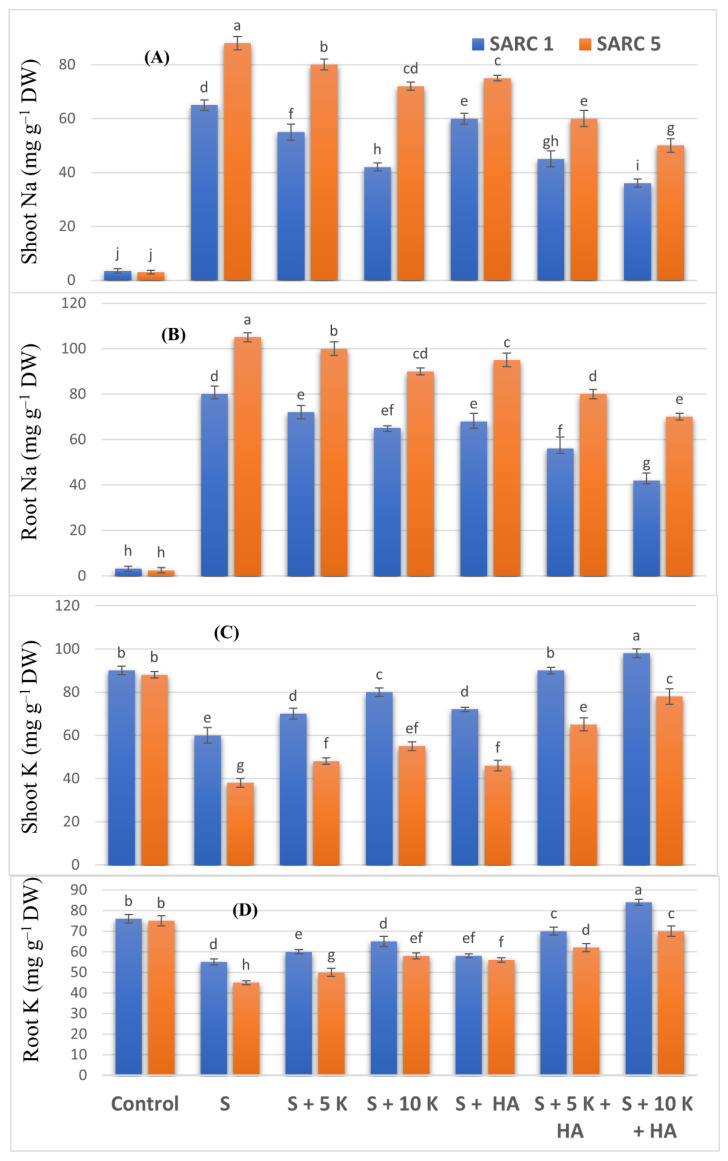
Effect of potassium (5, 10 mM K) and humic acid (HA, 2 g kg^−1^) on shoot Na (**A**), root Na (**B**), shoot K (**C**) and root K (**D**) concentrations of salt tolerant (SARC 1) and salt sensitive (SARC 5) wheat genotypes exposed to exposed to 100 mM NaCl salt stress (S). DW, dry weight. For each trait, bar data with the same letter indicate no significant difference between treatments (*p* < 0.05).

Shoot and root Na contents in SARC 1 and SARC 5 increased significantly under salinity as compared to control treatment (Figure 2A,B). The application of K and HA alone as well as in combination significantly reduced Na accumulation in both genotypes. The lowest Na contents were noticed under the combined application of 10 mM K and HA. The comparison of genotypes indicated that SARC 1 maintained relatively lower contents of Na in shoot and root than SARC 5. Shoot and root K contents declined significantly under saline conditions (Figure 2C,D). The application of K and HA alone as well as in combination significantly increased K accumulation in both genotypes. The highest K contents were found under the combined application of 10 mM K and HA. SARC-1 accumulated relatively higher contents of K in shoots and roots as compared to SARC 5.

**Figure 3:**
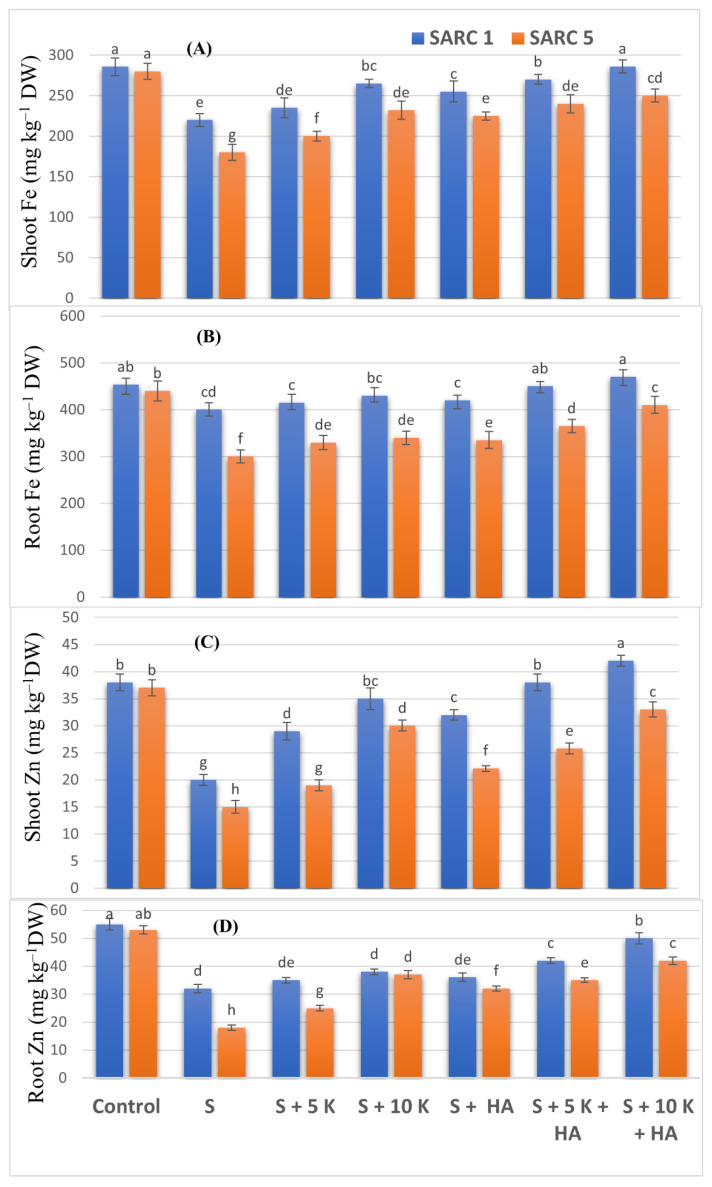
Effect of potassium (5, 10 mM K) and humic acid (HA, 2 g kg^−1^) on shoot Fe (**A**), root Fe (**B**), shoot Zn (**C**) and root Zn (**D**) of salt tolerant (SARC 1) and salt sensitive (SARC 5) wheat genotypes exposed to 100 mM NaCl salt stress (S). DW, dry weight. For each trait, bar data with the same letter indicate no significant difference between treatments (*p* < 0.05).

Shoot and root Zn and Fe contents decreased significantly under salinity stress (Figure 3A–D). The application of K and HA alone as well as in combination significantly increased shoot and root Zn and Fe accumulation in both genotypes. The highest Zn and Fe contents were found when 10 mM K and HA were applied together. SARC 1 accumulated more Zn and Fe as compared to SARC 5 in all the treatments.

### 2.4. Oxidative Stress Attributes

**Figure 4:**
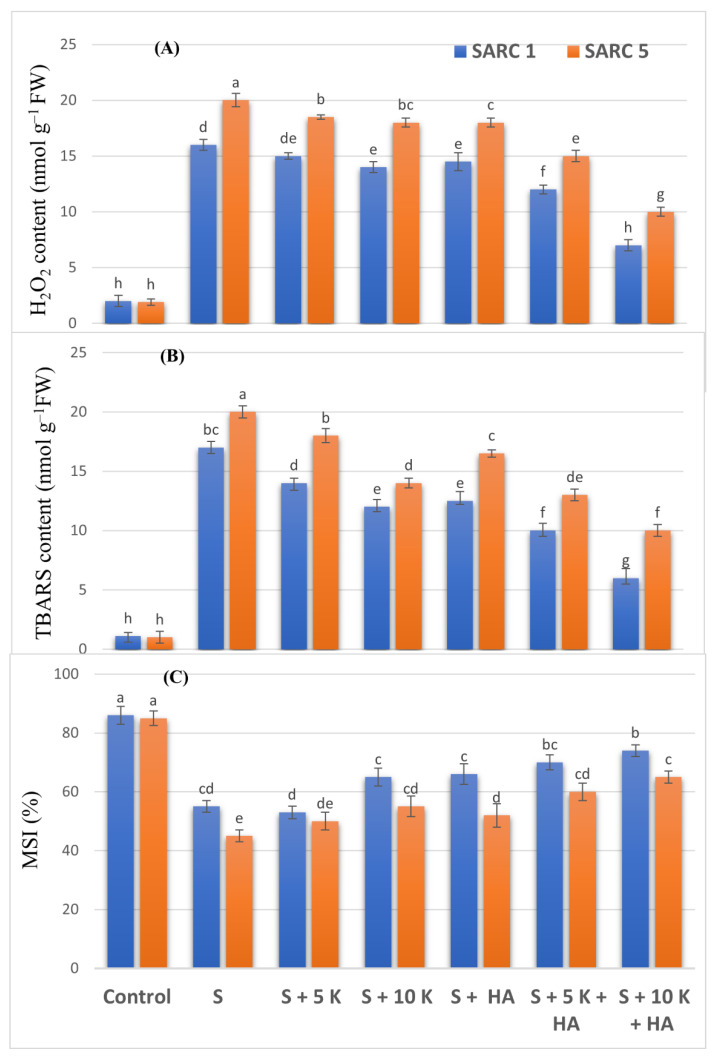
Effect of potassium (5, 10 mM K) and humic acid (HA, 2 g kg^−1^) on H_2_O_2_ contents (**A**), TBARS contents (**B**) and membrane stability index (**C**) of salt tolerant (SARC 1) and salt sensitive (SARC 5) wheat genotypes exposed to 100 mM NaCl salt stress (S). FW, fresh weight; TBARS, thiobarbituric acid reactive substances; MSI, membrane stability index. For each trait, bar data with the same letter indicate no significant difference between treatments (*p* < 0.05).

Due to salinity stress, the production of H_2_O_2_ and lipid peroxidation in TBARS was increased by 8-fold and 15-fold in SARC 1, and 10-fold and 20- fold in SARC 5, respectively as compared to control (Figure 4A,B). The application of K and HA significantly decreased the oxidative stress. In the combined application of 10 mM K and HA, H_2_O_2_ contents were 2.3-fold and 2-fold higher than saline treatment in SARC 1 and SARC 5, respectively. The contents of thiobarbituric acid reactive substances (TBARS) were decreased to 2.8-fold and 2-fold in SARC 1 and SARC 5, respectively when 10 mM K and HA were applied together. The MSI (membrane stability index) was decreased by 36% and 47% in SARC 1 and SARC 5, respectively in comparison to control (Figure 4C). The application of K and HA significantly improved the MSI and combined application of both K and HA was more effective than their single treatments. The MSI was enhanced by 34% and 44% than saline treatment in SARC 1 and SARC 5, respectively in the combined application of 10 mM K and HA.

### 2.5. Antioxidant Enzymes

**Figure 5:**
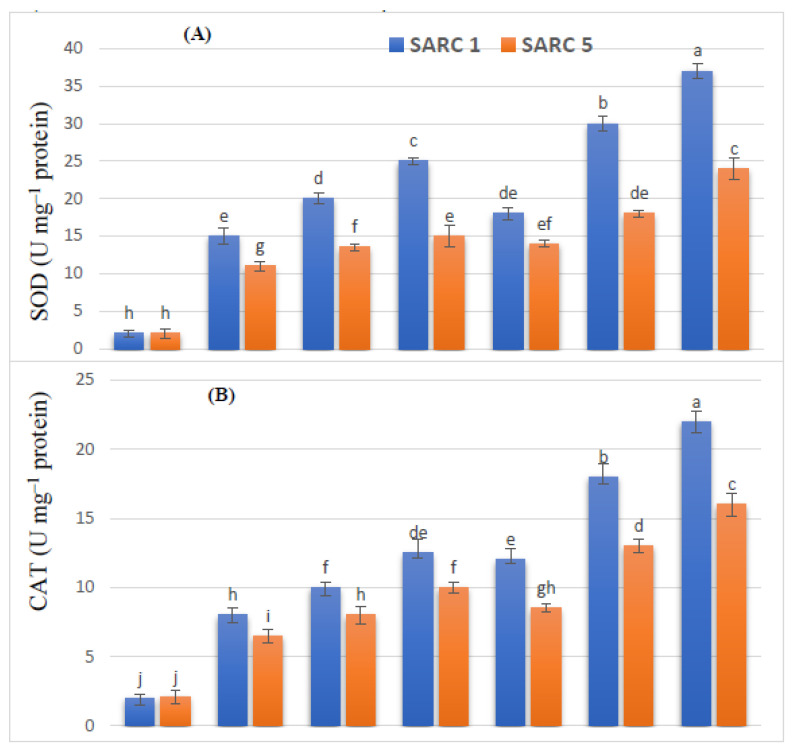

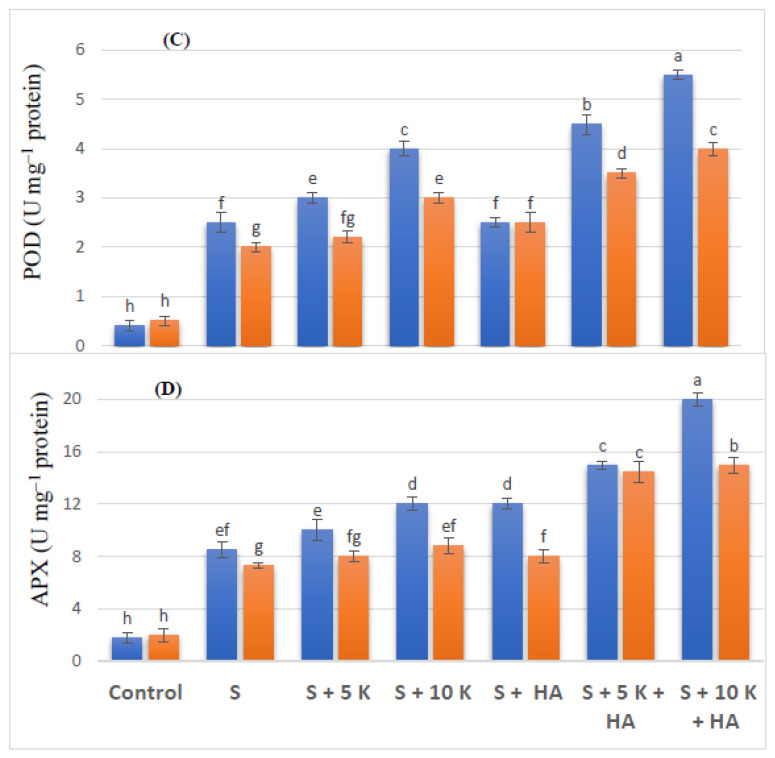
Effect of potassium (5, 10 mM K) and humic acid (HA, 2 g kg^−1^) on the activities of SOD (**A**) CAT (**B**), POD (**C**) and APX (**D**) of salt tolerant (SARC 1) and salt sensitive (SARC 5) wheat genotypes exposed to 100 mM NaCl salt stress (S). For each trait, bar data with the same letter indicate no significant difference between treatments (*p* < 0.05).

Salinity induced oxidative stress was ameliorated by over expression of antioxidant enzymes. The activities of all the enzymes (APX, CAT, POD, SOD) were increased under the application of K and HA and more so under their combined application (Figure 5). The enzymatic activities of APX, CAT, POD, and SOD were also increased by 1.7-fold, 1.6-fold, 1.6-fold and 1.4-fold in SARC 1, and by 1.4-fold, 1.5-fold, 1.5-fold and 1.2-fold in SARC 5 under the application of 10 mM K as compared to saline treatment. While the combined application of 10 mM K and HA, the activities of APX, CAT, POD, and SOD were also increased by 2.5-fold, 2.8-fold, 2.2-fold and 2.4-fold in SARC 1, and by 2.2-fold, 2.5-fold, 2.-fold and 2.1-fold in SARC 5as compared to saline treatment.

### 2.6. Multivariate Analyses

**Figure 6:**
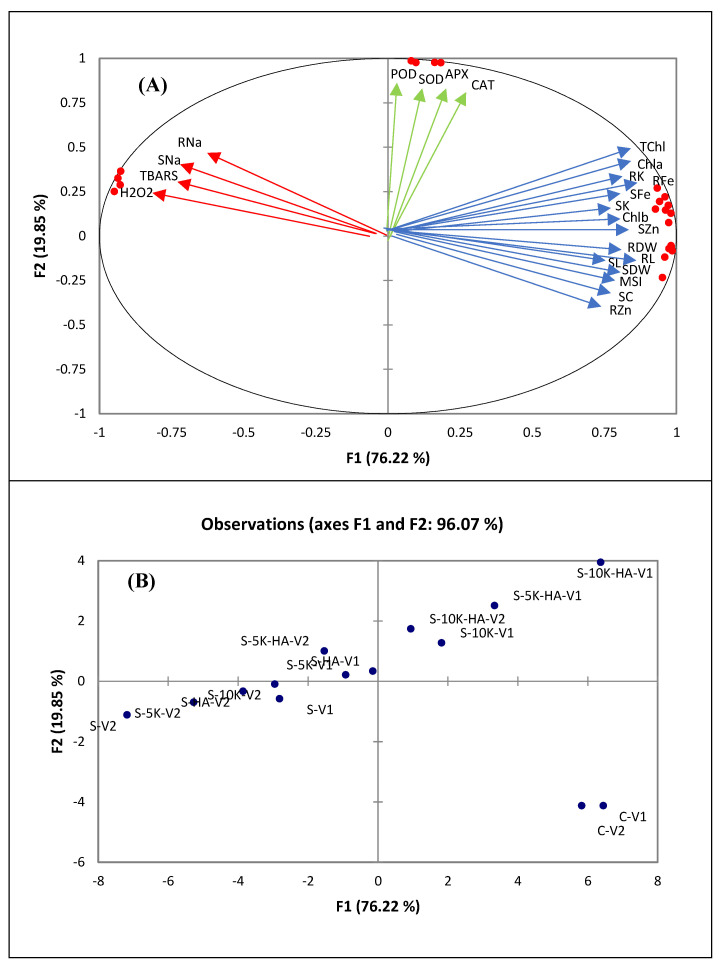
Principal component analysis of (**A**) response variables and (**B**) treatments and wheat genotypes exposed to 100 mM NaCl salt stress (S) supplemented with/without potassium (5, 10 mM K) and humic acid (HA, 2 g kg^−1^). V1, SARC 1; V2, SARC 5.

The relationship among different treatments and the resultant response variables was estimated through principal component analysis (PCA) as shown in Figure 6. It was depicted through PCA that thirteen factors (F1 to F13) contributed to the total variability, nevertheless, the major contribution was from only four factors. These four factors contributed 76%, 20%, 1.4% and 0.76% variability, respectively. As demonstrated in Figure 6A, there were three groups of the response variables (a) plant growth and physiological attributes were grouped closer to each other (b) root and shoot Na and oxidative stress attributes (H_2_O_2_, TBARS) and (c) antioxidant enzymes (SOD, CAT, APX, POD). Growth and physiological attributes were clustered opposite to oxidative stress attributes and Na contents in shoot and root. The response of different treatments and genotypes was also illustrated by PCA (Figure 6B). Both genotypes under salt stress treatment in the absence of K and HA was placed in negative X and Y axis. On the other hand, when K and HA were applied in combination under salinity stress, both genotypes were clustered in positive X and Y axis. Pearson correlation matrix depicted negative correlation of shoot and root Na contents and oxidative stress attributes with growth and physiological attributes of both genotypes (Table 3).

SL, shoot length; RL, root length; SDW, shoot dry weight; RDW, root dry weight; SNa, shoot Na; RNa, root Na; SK, shoot K; RK, root K; H_2_O_2_, Hydrogen peroxide; TBARS, thiobarbituric acid reactive substances; MSI, membrane stability index; SOD, superoxide dismutase; CAT, catalase; POD, peroxidase; APX, ascorbate peroxidase; SFe, shoot iron; RFe, root iron; SZn, shoot zinc; RZn, root zinc; Chla, chlorophyll a; Chlb, chlorophyll b; TChl, total chlorophyll; SC, stomatal conductance.

## 3. Discussion

**Figure 7:**
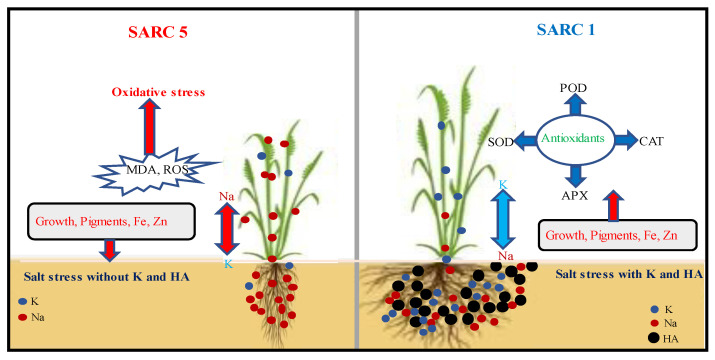
Plant growth, nutrient uptake, pigments, and oxidative stress attributes of salt tolerant (SARC 1) and salt sensitive (SARC 5) wheat genotypes exposed to 100 mM NaCl with and without potas-sium (K) and humic acid (HA). Plants supplemented with combined K and HA showed better salt tolerance and biomass production than without K and HA. Greater salt tolerance of SARC 1 than SARC 5 is mainly attributed to limited uptake of Na and higher activities of antioxidants.

The current investigation found that salinity decreased shoot and root growth of both genotypes (SARC 1 and SARC 5). Salt induced damage to plant growth is attributed to osmotic pressure, ionic toxicity, ionic imbalance, limited K uptake, and limitations to stomatal conductance and chlorophyll biosynthesis [7,33]. Supplementation of crops with mineral nutrients has shown very promising results in lessening the salt induced damages in crop plants. In our study, plant growth of both wheat genotypes was improved when external K was applied under the salt stress conditions (Figure 7). Among various nutrients, K has a well proven role in improving the growth of plant as well as physiology in salt stress situations [18,34,35]. In line with our current study, exogenous application of K has shown ameliorative effects on salt stress in wheat under soil culture conditions [33]. According to them [33], the main mechanisms of K induced enhancement in plant growth and yield included; enhanced accumulation and assimilation of different nitrogenous compounds, upregulation of antioxidant enzymes accompanied by limited uptake of Na and decreased Na: K ratio. Similarly, under solution culture conditions, Gul et al. [8] found that elevated K supply (8 mM) to salt stressed wheat seedlings reduced the Na accumulation in plant tissues and cytosol, improved plant water status and photosynthetic attributes leading to increased plant growth.

Our results indicated that the combined application of 10 mM K and HA was the best treatment for increasing salt tolerance of both genotypes (Figure 1) and demonstrated in Figure 7. Humic acid plays crucial role in increasing plant tolerance against several environmental stresses including drought and salinity [22,23,24,25]. Humic acid improves crop salt tolerance through the activation of numerous physiological and biochemical processes such as improved water relations, stomatal conductance, and activation of the antioxidant enzymes (CAT, POD and SOD) to counter the deleterious effects of ROS [25,26]. In accordance with our results, Ali et al. [25] found that HA application improved salt tolerance and biomass production by sorghum under salinity stress by increasing various salt tolerance indices, decreasing the generation of ROS and enhanced activities of antioxidants. Additionally, HA has been stated to increase plant growth by lowering the pH and increasing the nutrient status of the soils [27,29,36]. Wheat genotype SARC 1 was relatively more tolerant to salinity than SARC 5 particularly under the combined application of K and HA. Greater salt tolerance of SARC 1 was related to K and HA induced positive effects on stomatal conductance, photosynthetic process, osmotic adaption, ion homeostasis and activation of many enzymes and limited accumulation of Na and increased availability of essential nutrients [9,27,29].

Leaf pigments and stomatal conductance were decreased in both genotypes under salinity due to degradation of chlorophyll molecules caused by overproduction of ROS and replacement of K by Na ions and up-regulation of chlorophyllase activity [3,37]. These physiological changes also occur in response to other abiotic stress in wheat including waterlogging [38] and heat stress [39]. Salt induced damage to pigments was relieved under the application of K alone or in combination with HA. Enhanced provision of nitrogen to chlorophyll synthesis pathway along with replacement of Na by K might have contributed to increased chlorophyll biosynthesis under external K supply [33]. Additionally, external K and HA provide tolerance against oxidative stress and protect these macromolecules form the damaging effects of ROS.

Under normal growth conditions, ROS formation and detoxification in plants is maintained in balance. However, this balance is disturbed under stressful conditions. The production of ROS surpasses their detoxification resulting in oxidative burst, damage to macromolecules, cell membranes and ultimately cell death [37]. Under salinity stress situations, oxygen becomes the terminal electron acceptor rather than CO_2_ during photosynthesis. This shift results in the overproduction of ROS beyond the mitigation potential of plants [18]. In this study. both wheat genotypes suffered from oxidative stress due to excessive generation of H_2_O_2_ and TBARS leading to decline in the stability of the cell membranes. Similarly, many other studies have reported salt induced excessive production of ROS [18,33]. Closure of stomata due to dissociation of electron transport chain is the main reason for salt induced oxidative stress [37,38,39,40]. Lipid peroxidation activates K efflux channels causing the leakage of K [40]. Therefore, plants suffer from K deficiency under salinity stress conditions. The external supply of K along with HA ameliorated the oxidative stress by limiting the generation of H_2_O_2_. It has been reported that excessive ROS formation causes the over activation of plasma membrane K and Ca permeable cation channels such as GORK, SKOR and annexins. It results in excessive outflow of K from these channels and ultimately leading to apoptosis [41,42]. When H_2_O_2_ is converted into hydroxyl radicals, the resultant ROS become even more toxic. Hence, to safeguard the cell from toxic effects of H_2_O_2_, its conversion into less toxic forms should be done efficiently. Nature has gifted a strong defense system to plants in the form of antioxidant enzymes and non-enzymes [3,21]. Amongst numerous antioxidant enzymes, SOD is regarded as the most crucial one due to its major role in the conversion of superoxide radicals to H_2_O_2_ and oxygen [10,41]. SOD was overactivated under salinity and K supplementation along with HA caused even higher activity of SOD. Salinity induced overproduction of O_2_- in the cells and interaction between SOD and Na are the main reasons for overactivation of SOD [41]. Increased activity of SOD under salt stress with exogenous application of K [40,42,43] and HA [44] is well documented. SOD activity was more in SARC 1 than SARC 5 which is in line with its better defense against ROS and greater biomass production.

The enzymatic activities of APX, CAT and POD were also enhanced in both genotypes under salt stress. These enzymes catalyze the conversion of H_2_O_2_ into water and oxygen [10,14]. The activation of these enzymes under salinity has been observed in the past [3,45]. Potassium and HA addition further increased the activities of these enzymes that was quite helpful in reducing oxidative stress due to maintenance of adequate amount of NADP for optimal electron supply [25,41]. Over activation of antioxidants and resultant reduction in ROS induced membrane damage under K and HA supplementation is an indication of the ameliorating role of both amendments under salt stress conditions. Potassium down-regulate the activity of NADPH oxidases to decrease the level of ROS in the cells [41,46]. Likewise, HA is involved in various abiotic stress-related genes encoding that regulate the redox status of the cells [32]. The better salt tolerance of SARC 1 than SARC 5 is partly attributed to higher activities of these enzymes under salt stress which were further enhanced under the external supply of both K and HA.

Under saline treatment, shoot and root Na concentrations were increased, while K concentrations decreased significantly in both genotypes. Increased accumulation of Na and decreased uptake of K under salinity has been well documented [1,7]. The application of K and HA alone as well as in combination significantly reduced Na and increased K contents in both genotypes. Under salinity stress, K is leaked through the K efflux channels [40,47]. The exogenous application of K significantly improves K accumulation in the cells leading to better growth under saline conditions [48,49]. The comparison of genotypes indicated that SARC 1 maintained relatively lower Na and higher K concentrations in shoot and root than SARC 5. Higher growth and biomass production by SARC 1 is related to its better ionic composition in terms of lower Na and higher K contents [50].

Salt stress limited the uptake of Zn and Fe in both genotypes as observed by ref. [51]. It has been reported that under saline conditions the solubility of these micronutrients is decreased leading to their deficiency in plants [27,52,53]. HA alone or in combination with K significantly improved the concentrations of both Zn and Fe. Our results are in agreement with the outcomes of Khaled and Fawy [27] and Mohamed [44] who found an increase in the concentrations of micro-nutrients by the application of HA under salinity stress. Better growth and nutrient uptake due to HA application might be due to the better development of root systems and membrane permeability [54,55]. Due to the presence of both hydrophobic and hydrophilic sites, the surface of humic substances is very active [56]. Consequently, the humic substances can easily interact with phospholipid structures of the cell membranes and act as carriers of nutrients. The availability and uptake of Fe and Zn are increased due to chelation of these nutrient with HA [27]. On the other hand, K is also reported to increase the uptake of micronutrients in wheat under salt stress conditions due improvement in water relations [9]. SARC 1 accumulated more Fe and Zn than SARC 5. Lower genetic potential for Fe and Zn uptake and higher cellular Na might be the reasons for limited accumulation of Zn and Fe by SARC 5. Contrarily, greater potential for Zn and Fe uptake along with lower tissue Na contents might be the contributing factors for more Fe and Zn accumulation in SARC 1.

The relationship of different independent (treatments and both genotypes) and response variables was explained through a multivariate analysis (Figure 6). This data analysis technique provides very reliable information regarding the correlation and covariance of different treatments with the resultant response variables. In the case of current study, the PCA exhibited negative correlations of Na concentration and oxidative stress attributes (H_2_O_2_ and lipid peroxidation) against plant growth and physiological variables. That was in line with the obtained results as the genotype with higher Na (SARC 5) in shoot and root produced lower biomass and showed more decline in physiological attributes than SARC 1 genotype. The treatments of salt stress with and without the addition of K and HA were also separated by PCA. When K and HA were applied together under salinity stress, the treatments were clustered in positive X and Y axis. This is an indication that these treatments were better for plant growth under salt stress conditions for both genotypes. As discussed earlier, the better growth both genotypes under the concurrent application of K and HA could be due to multiple benefits such as limited uptake of Na, higher uptake of K, Zn and Fe, improved pigments and water status, and better protection against oxidative stress. On the hand, the salt stress treatments without K and HA or their sole application were clustered in negative X and Y axis. This indicated more damaging effects of salinity on plant growth and physiological attributes of both genotypes.

## 4. Materials and Methods

### 4.1. Growth Conditions and Treatment Application

The current study was carried out at the Department of Environmental Sciences, COMSATS University Islamabad, Vehari Campus. The average range of minimum and maximum temperature was 15–29 °C, and relative humidity was 47–75% during the experimental period. Two wheat genotypes [SARC 1 (salt-tolerant) and SARC 5 (salt-sensitive)] were grown up in pots (diameter, 15 cm; depth, 10 cm) containing acid washed sand (500 g) and three seeds of both wheat genotypes were then sown in each pot. The pots were irrigated with Hoagland’s solution (half strength) after seed germination, and one plant was retained in each pot. Salt stress (100 mM NaCl) was applied after one week of seed germination by dissolving the calculated amount of NaCl in distilled water, with appropriate controls. Humic acid (0, 2 g kg^−1^; Dalian Vic Co., Ltd., Dalian, China) containing 55% C, 0.01% P, 4.87% N, 0.22% Mg, 0.50% Ca, and 11.21% K with pH 7.08 was applied to the substrate before sowing of seeds. The treatments of K (0, 5, 10 mM K) were applied using K_2_SO_4_ salt one week after the salinity treatment. All the treatments were replicated four times and the experimental design was completely randomized.

### 4.2. Harvesting and Elemental Analysis

After the completion of six weeks of treatment, all plant samples were then harvested and carefully separated into roots and shoots for determination of growth data. Samples were washed with distilled water and dried with blotting paper. Root and shoot lengths were measured using a ruler. Subsequently, plants were kept in oven at 65 °C until constant dry weight to measure the growth response. For elemental analysis (Na, K, Fe, Zn), oven dried plant samples were acid digested (HNO_3_:HClO_4_; 2:1). The concentrations of Na and K ions in roots and shoots were determined using flame photometer (BWB-XP5). Whereas, the concentrations of Fe and Zn were measured using atomic absorption spectrophotometer (PerkinElmer Model: PinAAcle900F Inc., Waltham, MA, USA)

### 4.3. Stomatal Conductance and Chlorophyll Contents

Leaf porometer (Decagon Devices, Pullman, WA, USA) was used for measuring stomatal conductance of the second mature leaf from the top on a full sunny day one day before harvesting. Chlorophyll contents (Chl-a, Chl-b and total chlorophyll (Chl a + b) were estimated as mentioned by Lichtenthaler [57]. Briefly, leaf samples (0.5 g) were fully frozen in liquid nitrogen followed by grinding in 80% (*v*/*v*) solution of hydro-acetone. The material obtained was completely centrifuged at 3000× *g* for about 10 min. The absorbance values of the obtained supernatant were measured at 663.2, 646.8 and 470 nm using a UV–Vis spectrophotometer (Lambda 25, PerkinElmer, Inc. Los Angeles, CA, USA) for Chl-a, Chl-b and total chlorophyll contents.

### 4.4. Oxidative Stress Attributes

The contents of hydrogen peroxide (H_2_O_2_) were estimated using the method of Islam et al. [58]. For this purpose, leaf samples taken from each plant (0.5 g) were frozen in liquid nitrogen and then fully homogenized in 0.1% trichloroacetic acid. Following homogenization, centrifugations of all the mixtures were completed at 12000× *g* for 20 min. Subsequently, one mL of the 10 mM potassium phosphate buffer was combined with 1 mL of plant extract and 1 mL of the (2 M) potassium iodide. The absorbances of the mixtures were noted at 390 nm with a UV–Vis spectrophotometer. Thiobarbituric acid reactive substances (TBARS) equivalent to malondialdehyde (MDA) were measured as explained by Hodges et al. [59]. Hydro-alcoholic solution (80/20: *v*/*v*) was applied for homogenization of leaf samples (0.5 g). After homogenization, 1 mL solution of TBA was introduced into the samples which were then incubated at about 95 °C. Afterwards, centrifugation of the homogenate was done for 10 min at 12,000× *g*. The absorbance of supernatant was then determined at 532 nm using UV–Vis spectrophotometer and TBARS contents were mentioned in nmol g^−1^ on fresh weight basis. Membrane stability index (MSI) was determined as described by Sairam et al. [60].

### 4.5. Antioxidant Enzymes Activities

For the quantification of antioxidant enzyme activities (SOD, CAT, APX, POD), all the leaf samples (0.5 g) were ground in potassium phosphate buffer (2 mL) of 0.1 M strength (pH 7) followed by centrifugation at 10,000× *g* for 20 min at 4 °C. The supernatant of these samples was used for determination of antioxidant enzymes. The activity of SOD was determined as outlined by Gupta et al. [61]. One unit of the SOD enzymes was described as the total amount of SOD needed for 50 % reduction in nitro blue tetrazolium. Catalase was determined as described by Aebi [62]. Moreover, the activity of CAT was expressed in μM of hydrogen peroxide reduced min^−1^ mg^−1^ protein. Furthermore, the activity of APX was assayed as mentioned by Amako et al. [63], and was stated as μM of ascorbate oxidized min^−1^ mg^−1^ protein. The methodology described by Hemeda and Klein [64] was followed for POD measurement which was expressed as µM guaiacol oxidized min^−1^ mg^−1^ protein.

### 4.6. Statistical Analysis

The statistical software Statistix 8.1 was used for data analysis through two-way analysis of variance (ANOVA). Data of treatments and genotypes were compared using least significant difference test at about 5% probability level [65].

## 5. Conclusions

Plant growth and physiological attributes of both wheat genotypes (SARC 1 and SARC 5) were adversely affected under salinity stress. Supplementation of K and HA relieved the oxidative stress and improved the growth and physiological attributes in wheat. The combined application of HA and 10 mM K was the most effective treatment in alleviating salt stress and increasing nutrient uptake in both genotypes. Genotype SARC 1 showed better growth and greater tolerance to NaCl stress than SARC 5 with supplementation of K and HA, alone or in combination. Nevertheless, further investigations are required to validate the interactive effects of K and HA in alleviating wheat tolerance to salt stress grown in saline soils involving more wheat genotypes and under controlled and field conditions, and to explore the underlying molecular mechanisms.

## Figures and Tables

**Table 1:** Effect of potassium (5, 10 mM K) and humic acid (HA, 2 g kg^−1^) on shoot length, root length, shoot dry weight and root dry weight of salt tolerant (SARC 1) and salt sensitive (SARC 5) wheat genotypes exposed to 100 mM NaCl salt stress (S).

Treatments	Shoot Length (cm)		Root Length (cm)		Shoot Dry Weight (g plant ^−1^)		Root Dry Weight (g plant ^−1^)	
	SARC 1	SARC 5	SARC 1	SARC 5	SARC 1	SARC 5	SARC 1	SARC 5
Control	49.0 a	47.3 a	47.9 a	45.0 a	0.46 a	0.45 ab	0.15 a	0.15 a
S	32.5 efg	27.0 h	31.2 cde	25.0 h	0.30 ef	0.23 h	0.10 def	0.08 f
S + 5 K	34.2 def	28.0 h	34.3 c	27.0 gh	0.33 de	0.26 gh	0.12 bcd	0.09 ef
S + 10 K	39.0 c	31.0 g	39.0 b	30.0 efg	0.38 c	0.27 fg	0.13 abc	0.10 def
S + HA	35.0 de	28.0 h	34.2 c	28.0 fgh	0.35 cd	0.26 gh	0.11 cd	0.09 ef
S + 5 K + HA	43.0 b	32.0 fg	40.1 b	31.0 def	0.33 b	0.30 ef	0.13 abc	0.10 def
S + 10 K + HA	48.2 a	36.0 d	46.0 a	34.0 cd	0.44 ab	0.33 de	0.15 a	0.11 cd

Data presented are the mean values of four replications. The mean multiple comparison tests were performed for both genotypes for each trait. The values followed by same letters indicate non-significant difference at 5% probability level.

**Table 2:** Effect of potassium (5, 10 mM K) and humic acid (HA, 2 g kg^−1^) on Chlorophyll a, Chlorophyll b, total chlorophyll and stomatal conductance of salt tolerant (SARC 1) and salt sensitive (SARC 5) wheat genotypes exposed to 100 mM NaCl salt stress (S).

Treatments	Chlorophyll a (µg g^−1^)		Chlorophyll b (µg g^−1^)		Total Chlorophyll (µg g^−1^)		Stomatal Conductance (mmol m^−2^ s^−1^)	
	SARC 1	SARC 5	SARC 1	SARC 5	SARC 1	SARC 5	SARC 1	SARC 5
Control	290 b	285 b	180 b	175 b	470 b	460 b	440 a	435 a
S	230 ef	200 g	140 d	120 f	370 e	320 g	350 de	295 f
S + 5 K	245 e	220 f	145 d	125 ef	390 d	345 f	370 d	320 e
S + 10 K	280 bc	235 ef	160 c	132 ef	440 c	367 ef	405 b	340 de
S + HA	265 d	255 d	146 d	136 e	411 d	391 e	365 d	325 e
S + 5 K + HA	295 b	255 d	172 bc	145 d	467 b	410 e	395 bc	350 de
S + 10 K + HA	320 a	272 c	192 a	160 c	512 a	430 c	438 a	360 d

Data presented are the mean values of four replications. The mean multiple comparison tests were performed for both genotypes for each trait. The values followed by same letters indicate non-significant difference at 5% probability level.

**Table 3:** Pearson correlation matrix of various response variables of wheat genotypes exposed to 100 mM NaCl salt stress supplemented with/without potassium (5, 10 mM K) and humic acid (HA, 2 g kg^−1^). Values in bold represent the significant difference at 0.05% probability level.

Traits	SL	RL	SDW	RDW	SNa	RNa	SK	RK	H_2_O_2_	TBARS	MSI	SOD	CAT	POD	APX	SFe	RFe	SZn	RZn	Chla	Chlb	TChl
RL	**0.99**	**1.00**																				
SDW	**0.99**	**0.99**	**1.00**																			
RDW	**0.98**	**0.99**	**0.98**	**1.00**																		
SNa	**−0.94**	**−0.95**	**−0.93**	**−0.94**	**1.00**																	
RNa	**−0.94**	**−0.94**	**−0.94**	**−0.93**	**0.99**	**1.00**																
SK	**0.95**	**0.94**	**0.96**	**0.95**	**−0.86**	**−0.84**	**1.00**															
RK	**0.93**	**0.92**	**0.90**	**0.91**	**−0.86**	**−0.84**	**0.94**	**1.00**														
H_2_O_2_	**−0.93**	**−0.92**	**−0.91**	**−0.90**	**0.97**	**0.97**	**−0.85**	**−0.89**	**1.00**													
TBARS	**−0.94**	**−0.94**	**−0.92**	**−0.92**	**0.97**	**0.97**	**−0.87**	**−0.91**	**0.98**	**1.00**												
MSI	**0.95**	**0.94**	**0.94**	**0.91**	**−0.96**	**−0.97**	**0.88**	**0.88**	**−0.96**	**−0.98**	**1.00**											
SOD	0.11	0.09	0.12	0.11	0.17	0.21	0.33	0.28	0.14	0.11	−0.09	**1.00**										
CAT	0.12	0.09	0.12	0.09	0.15	0.18	0.35	0.33	0.10	0.05	−0.04	**0.97**	**1.00**									
POD	0.02	0.00	0.02	0.02	0.24	0.29	0.25	0.23	0.21	0.17	−0.17	**0.98**	**0.97**	**1.00**								
APX	0.02	0.00	0.03	0.00	0.22	0.26	0.27	0.25	0.16	0.13	−0.12	**0.95**	**0.98**	**0.96**	**1.00**							
SFe	**0.92**	**0.94**	**0.94**	**0.93**	**−0.88**	**−0.86**	**0.95**	**0.94**	**−0.85**	**−0.91**	0.92	0.22	0.25	0.15	0.17	**1.00**						
RFe	**0.90**	**0.91**	**0.94**	**0.92**	**−0.82**	**−0.81**	**0.97**	**0.86**	**−0.80**	**−0.79**	0.83	0.32	0.30	0.21	0.23	**0.91**	**1.00**					
SZn	**0.90**	**0.90**	**0.90**	**0.91**	**−0.81**	**−0.79**	**0.95**	**0.92**	**−0.79**	**−0.86**	0.85	0.34	0.37	0.27	0.26	**0.96**	**0.88**	**1.00**				
RZn	**0.93**	**0.93**	**0.91**	**0.92**	**−0.93**	**−0.92**	**0.90**	**0.94**	**−0.93**	**−0.97**	0.94	0.02	0.07	−0.03	−0.02	**0.95**	**0.84**	**0.90**	**1.00**			
Chla	**0.88**	**0.89**	**0.89**	**0.87**	**−0.78**	**−0.76**	**0.93**	**0.94**	**−0.78**	**−0.82**	0.84	0.40	0.43	0.33	0.35	**0.96**	**0.88**	**0.93**	**0.88**	**1.00**		
Chlb	**0.97**	**0.96**	**0.96**	**0.94**	**−0.88**	**−0.87**	**0.97**	**0.98**	**−0.89**	**−0.90**	0.90	0.29	0.31	0.21	0.22	**0.94**	**0.92**	**0.92**	**0.92**	**0.96**	**1.00**	
TChl	**0.92**	**0.92**	**0.92**	**0.90**	**−0.82**	**−0.81**	**0.95**	**0.97**	**−0.83**	**−0.86**	0.87	0.36	0.39	0.29	0.31	**0.96**	**0.90**	**0.93**	**0.90**	**0.99**	**0.98**	**1.00**
SC	**0.98**	**0.99**	**0.98**	**0.99**	**−0.94**	**−0.93**	**0.95**	**0.92**	**−0.90**	**−0.92**	0.92	0.12	0.11	0.03	0.03	**0.95**	**0.92**	**0.91**	**0.93**	**0.89**	**0.95**	**0.92**

## Data Availability

All data are included in this paper or upon requested for the correspondence authors.
